# Investigation of an Organogel by Micro-Differential Scanning Calorimetry: Quantitative Relationship between the Shapes of the Thermograms and the Phase Diagram

**DOI:** 10.3390/gels7030093

**Published:** 2021-07-14

**Authors:** Duncan Schwaller, Elliot Christ, Mélanie Legros, Dominique Collin, Philippe J. Mésini

**Affiliations:** Institut Charles Sadron, Université de Strasbourg, CNRS UPR22, 23 Rue du Loess, 67000 Strasbourg, France; duncan.schwaller@etu.unistra.fr (D.S.); elliot.christ@gmail.com (E.C.); melanie.legros@ics-cnrs.unistra.fr (M.L.); dominique.collin@ics-cnrs.unistra.fr (D.C.)

**Keywords:** organogels, phase diagrams, micro differential scanning calorimetry

## Abstract

The phase diagrams of organogels are necessary for applications and fundamental aspects, for instance to understand their thermodynamics. Differential scanning calorimetry is one of the techniques implemented to map these diagrams. The thermograms of organogels upon heating show broad endotherms, increasing gradually to a maximum, at a temperature $T_{\max}$, and decreasing back to the baseline, sometimes 10 °C above. This broadening can lead to uncertainty in determining the molar enthalpies and the melting temperatures $T_{m}$ of the gels. Herein, we have measured the thermograms of the 12-hydroxystearic acid/nitrobenzene gels for weight fractions ranging from 0.0015 to 0.04. Compared with transition temperatures measured by other techniques, the inflection points of the thermograms provide a measurement of $T_{m}$ with less bias than $T_{\max}$. The phase diagram explains why the molar melting enthalpies derived from the thermograms for samples of low concentration are lower than expected. The shapes of the heat flows below the peak correlate quantitatively with the diagrams: after suitable correction and normalization, the integral curves superimpose with the phase diagram in their ascending branch and reach a plateau when the gel is fully melted. The shape of the thermograms upon cooling is also qualitatively explained within the frame of the diagrams.

## 1. Introduction

Organogelators are small molecules that are able to gel organic solvents at low concentrations, typically a few weight percent [1,2,3,4,5,6]. These compounds self-assemble into fibrillar aggregates through non-covalent bonds. The aggregates form a 3D network endowing the mixture with its mechanical properties. The first step of the characterization of such systems should be the determination of their *c*-*T* phase diagrams. These diagrams are powerful tools to compare the efficiency of the gelators. Indeed, they represent the thermal stabilities of the gels and their domains of existence in a full range of concentrations. A mere minimal gel concentration cannot provide this information. Moreover, diagrams provide the experimental basis to understand the thermodynamics of gelation. Feng et al. [7] have mapped the full phase diagrams of triamide gelators and have modeled them by Hansen’s parameters in the frame of a regular model. The phase diagrams are essential for any application to tune the operating temperature and concentrations, and also to understand the properties of the gels. For instance, in gelator/polymer systems where the gelator is a clarifying agent, the phase diagrams help to understand why the variations of the optical properties do not vary monotonously with concentration [8,9,10].

In order to map the phase diagrams, the most popular techniques to determine phase transition temperatures are the tube inversion or falling spheres tests [11]. These techniques are easy to set up and necessitate small amounts of compounds. However, as pointed out in the literature [11], these simple techniques do not control the applied stress, and they may exceed the yield stress. It often leads to an apparent value of the sol–gel transition temperature lower than those measured by other techniques, and the difference can be as high as 10 °C [12]. This systematic error is avoided with a rheometer, since the stress can be adjusted to remain within the linear regime.

Another technique used to map phase diagrams is differential scanning calorimetry (DSC), which measures the enthalpy associated with the self-association of gelators molecules. The temperatures measured by this technique are only related to the thermodynamics of the systems. It can indicate the temperature for which all the solid phase has melted (called $T_{m}$ hereafter). The DSC experiment cannot measure the temperature where the system transforms into a sol (called $T_{\mathrm{GS}}$) because it is defined by rheology. The rheological transition may happen when there is still a solid fraction (that is below $T_{m}$) but not enough to connect into a network. Even if $T_{m}$ and $T_{\mathrm{GS}}$ have close values, they remain different quantities.

In DSC, upon cooling, the solidification is easily identified by a sharp peak in the thermograms, and the temperature of its maximum, which is easy to locate accurately and is often chosen as the transition temperature [11,13]. In terms of thermodynamics, the relevant temperature of the formation of the solid fraction should be the onset temperature [14]. However, as underlined by Toro-Vasquez [13], the measurement of the onset of the heat flow has a greater uncertainty, since the choice of the temperature for which the heat flow departs from the baseline is arbitrary. However, both methods of measurement yield close values, thanks to the sharpness of the peak during cooling.

The determination of the melting temperature during the heating phase is more delicate. The heat flows measured on organogels are low and the peaks may be very broad or even absent [15]. However, in most cases, the temperature of peak maximum, $T_{\max}$, is easy to locate and is usually taken in the community of organogels as the gel-to-sol temperature. Desvergnes et al. [16] have noticed a 5 °C gap with this value and the one measured by other techniques. They have also noticed a large difference in molar enthalpies between the heating and cooling phases. In other fields working with phase diagrams [14] or for some authors in the field of organogelators [17], the relevant temperature is the end of the peak, when the heat flow merges with the baseline. Since the peaks are broad, the gap between this temperature and $T_{\max}$ is often consequent. In these conditions, where to measure the transition temperature on the thermogram, and is the temperature of the peak relevant? The shape of the thermograms in other fields has been modeled and explained in relation with the phase diagram [18] but very seldom in the case of organogelator/solvent systems.

In this study, we have studied gels of 12-hydroxystearic acid (HSA) in nitrobenzene by micro-differential scanning calorimetry (microDSC). This system was chosen because its phase diagram is well known and has been mapped by two different groups with several techniques, over almost two decades of concentrations [12,19]. MicroDSC operates at low heating rates, and the thermogram are less impacted by heat transfer and kinetics, and we wondered if in these conditions they reflect only the equilibrium of the gels. We have tested different methods to measure the transition temperatures from the heat flows by comparing the obtained values with the phase diagram. The stretched shape of the heat flows was explained by correlating the integral curves with the phase diagrams.

## 2. Results and Discussion

### 2.1. Aspect of the Thermograms

**Figure 1:**
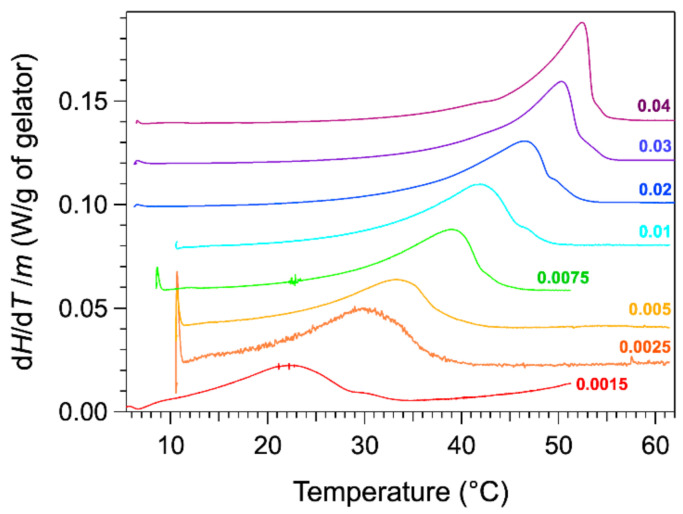
DSC thermograms of HSA/nitrobenzene gels for various weight fractions *W* from 0.0025 to 0.04 in HAS (endo up). Heating rate 0.1 °C/min. The curves were normalized to the masses *m* of the gelator HSA and staggered for clarity.

The thermograms were measured with a microcalorimeter. The apparatus measures the heat flow difference between the gel and the solvent as the reference. The advantage of the microcalorimeter is its sensitivity at low heating rates. The rate used in our studies is 0.1 °C/min. The low rates provide a lower signal, but they reduce the effects due to the heat transfer and kinetics. For instance, the observed peaks are not shifted to higher temperature values than actual temperatures. The thermograms of HSA/nitrobenzene gels were recorded for total weight fractions *W* comprised between 0.0015 and 0.04. The thermograms normalized to the mass of HSA are presented in Figure 1.

The thermograms for weight fractions *W* exhibit an endothermic peak, stretched out from low temperature, increasing slowly up to a maximum, and followed by steep decrease. The heat flow diminishes to the baseline only a few degrees after its maximal value. For samples at lower concentrations, the decrease after the peak is less abrupt and much slower. For the last curve, the heat flow reaches the baseline 10 °C above the temperature of the maximal flow. At the maximum of the peak $\;T_{\max}$, the thermal events are far to be finished, and the choice of the temperature of this maximum as $T_{m}$ is not obvious. This will be discussed later.

### 2.2. Measurements of the Molar Enthalpies

**Figure 2:**
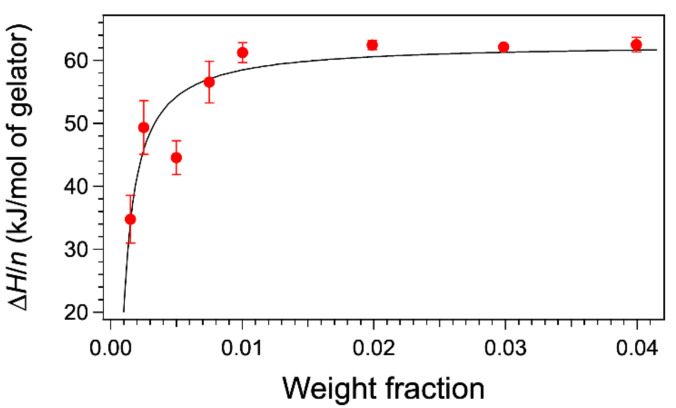
Apparent molar enthalpies as a function of weight concentration. The enthalpies ∆*H* are calculated by integrating the heat flows from 14 °C to a temperature above the complete melting of the gel. They are divided by *n*, the number of moles of gelator in the sample. Dashed line fit of the data with Equation (4) corresponding to the lever rule. The optimal parameters of the fit are $w_{l,1}$ = 0.00067 (solubility at 14 °C in wt % fraction); *L* = 62.7 kJ/mol (molar melting enthalpy).

From these thermograms, the enthalpies can be measured by integration, and one may wonder whether the change of shape of the curves with concentration affects the enthalpies. We have integrated the heat flows either with the software of the apparatus or home-made software. In both cases, the signal is integrated between two temperatures after a baseline is subtracted. This baseline is simply the straight line joining both points. As a consequence, at the temperatures chosen as the integration limits, the heat flow is set to zero. In this first part, we have followed this procedure, and we will discuss its accuracy later. For each thermogram, the curves were integrated from the same starting temperature 14 °C up to temperatures after the peaks, when the heat flows are equal to the baseline. The calculated enthalpy ∆*H* was normalized to the number of moles of gelator *n* in the sample to yield the molar melting enthalpies *L* (Figure 2). For weight fractions above 0.01, the values are the same with an average value of 62.1 ± 0.3 kJ/mol. However, the values diminish for lower concentrations. The thermograms at a rate of 0.25 °C/min afford the same values of ∆*H* within an error of 3%, thus showing no effect of the rate. The low concentrations are subjects to the errors in weighing low masses, and in the sensibility of the calorimeter, but these errors do not exceed 10% of total area and cannot explain a drop of 50% for *W* = 0.0015.

**Figure 3:**
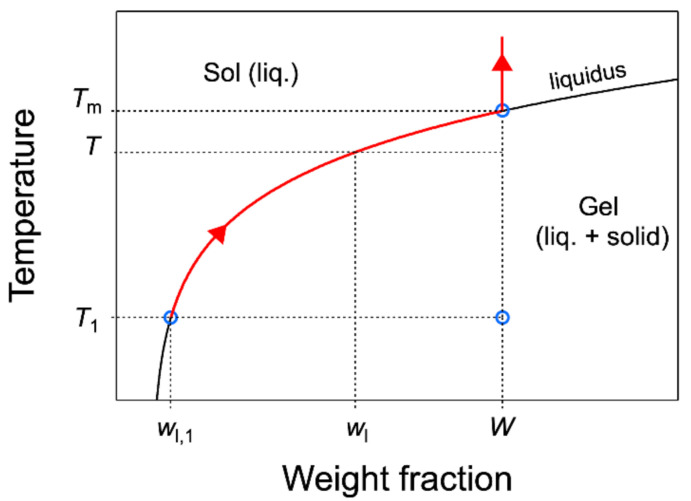
Schematic phase diagram, with the definition of the quantities used in equations. $T_{m}$ is the temperature where all the gelator is solubilized. $w_{l}$ is the weight fraction of the gelator in the liquid phase. Above $T_{m}$, $w_{l}\left(T\right)=W$. $T_{1}$ is the lower limit integration of the heat flow to calculate the enthalpy ∆*H*. The red part of the liquidus and the arrow indicate the evolution of the gelator fraction in the liquid phase when *T* increases.

The apparent lower values at low concentrations are easily explained by the solubility of the gelator. If the measured enthalpy is assumed to come only from the melting of the gel, the heat flow is given by Equation (1).

(1) $$\frac{dH}{dT}=-L\frac{dn_{s}}{dT}=L\frac{dn_{l}}{dT}$$

where *L* is the molar enthalpy of melting of the gel and d*n*_s_ is the number of moles of gelators transitioning from the solid state to the liquid phase, opposite to $dn_{l}$, the increment in the number of moles appearing in the liquid phase. If the heat flow is integrated between a temperature $T_{1}$ (chosen as low as possible) and a temperature *T*, the corresponding enthalpy is:

(2) $$∆H_{1}^{T}=L\left(n_{s,1}-n_{s}\right)=L\left(n_{l}-n_{l,1}\right)$$

where $n_{s}$ and $n_{s,1}$ are the number of moles of gelator in the solid phase at *T* and $T_{1}$; $n_{l}$ and $n_{l,1}$ are the same in the liquid phase. The integral $∆H_{1}^{T}$ increases with *T* and reaches a plateau when *T* is above the melting temperature of the gel $T_{m}$. This plateau, noted simply ∆H, is the value used above to derive molar enthalpy ($∆H_{1}^{T>T_{m}}=∆H$). When $T>T_{m}$, all the gelator is solubilized, there is no longer solid: $n_{s}$ = 0; the corresponding integral $∆H=Ln_{s,1}=L\left(n-n_{l,1}\right)$, with *n* representing the total number of moles of the gelator. There is always a proportion of the gelator soluble in the liquid phase even if it is small. It is related to the concentration $w_{l}$ of the gelator in the liquid, or solubility; it depends on the temperature only, and it is given by the gel-to-sol line in the phase diagram (the abscissa at a given *T*; see Figure 3).

When the total fraction *W* in the gelator is high, the fraction of the soluble fraction of the gelator is negligible before the solid fraction: *n* ∼ $n_{s,1}$ and *∆H* = *Ln*. For high *W* values, it is a good approximation to consider that all the gelator in the mixture transitions from the solid to the liquid phase. However, this approximation is no longer valid when *W* is in the same order of magnitude as the solubility of the gelator. The lever rule allows quantifying the weight fraction of the solid phase:

(3) $$\frac{m_{s,1}}{M_{t}}=\frac{W-w_{l,1}}{1-w_{l,1}}$$

where $m_{s,1}$ is the mass of the solid gelator at $T_{1}$ and $M_{t}$ is the total mass of the gel; $w_{l,1}$ is the solubility at $T_{1}$ ($w_{l,1}=w_{l}\left(T_{1}\right)$). From this equation, the number of moles of gelator *n*_s_ in the solid phase can be calculated and hence the quantity ∆*H*/*n* (Equation (4)).

(4) $$\frac{∆H}{n}=L\frac{1}{W}\frac{W-w_{l,1}}{1-w_{l,1}}$$

In this equation, ∆H is the integral of the heat flow between a low temperature $T_{1}$ and a temperature above the transition, and $w_{l,1}$ is the fraction of gelator in the liquid at $T_{1}$.

∆*H*/*n* = *L* only when W >> $w_{l,1}$. Figure 2 shows the fit of the values from Equation (4). This fit accounts for the variations of *∆H*/*n*, with a parameter $w_{l,1}$ in the expected order of magnitude (about half of 0.0015). The most important point is that the value of *L* can be given by DSC only if the concentration *W* is significantly higher than the solubility $w_{l}$ at low temperature. In conclusion, the phase diagram can explain the dependance of the integrals with concentrations.

### 2.3. Measurement of Transition Temperatures

As an introduction, it is necessary to define the melting temperature $T_{m}$ of organogels. For a binary system such as organogels, according to Gibb’s phase rule, at fixed pressure, the sol–gel equilibrium is monovariant, which means that the gel melts in a range of temperatures, not at a single temperature. Even at low temperatures, a fraction of the solid melts upon heating, and the fraction of melted solid increases with *T*. This fraction is given by the lever rule, as explained above. When the temperature reaches $T_{m},$ the liquidus composition is the total concentration *W*, and all the gelator is solubilized (Figure 3). In summary, the solid fraction dissolves gradually from low temperature up to $T_{m}$ where the dissolution is complete.

**Figure 4:**
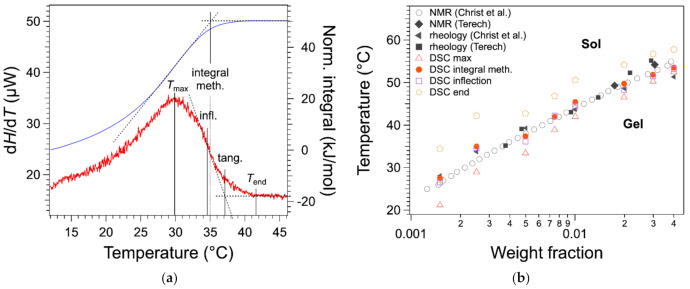
(**a**) Different measured temperatures on a thermogram (e.g., weight fraction *W* = 0.0025). Red: heat flow; blue: integral of the heat flow $∆H_{1}^{T}$. $T_{\max}$ is the temperature at the maximum of the endotherm, $T_{\mathrm{end}}$, at the end of the peak. The gap between both temperatures is about 12 °C. Two other temperatures measured on thermograms are the inflection point and the temperature by the tangent method [21]. The integral method measures the temperature at the intersection of the plateau and the tangent of the ascending branch at $T_{\max}$. Note that $T_{\mathrm{GS}}$ measured by rheology in this case is 34.7 °C. (**b**) Superimposition of the different *T* values with the *c*-*T* phase diagram of HSA/nitrobenzene measured by NMR, rheology, DSC, by us and others (Christ et al.: Ref. [19]; Terech: Ref. [12]). The errors of $T_{\max}$ and $T_{\mathrm{infl}}$ are 0.2 °C; the errors of $T_{\mathrm{end}}$ and *T* measured by the integral method are 0.5 °C. For DSC experiments, the uncertainties of the weight fractions *W* are less than 2.9%.

In previous work [20], we have considered $T_{m}$ as the temperature of the maximum $T_{\max}$ (Figure 4a), as practiced in earlier work or recommended in textbooks [11,13]. We have compared the $T_{\max}$ values with those obtained by other techniques. The binary *c*-*T* phase diagram of HSA/nitrobenzene provides a good comparison because it has been studied by two groups, each with different techniques and five sets of data with a good agreement [12,19]. There a bias between the measured $T_{\max}$ values temperature and the other measurements as shown in Figure 4b. All $T_{\max}$ values (red triangles) measured from DSC are lower than the values from other measurements.

For the highest two concentrations, there is a good agreement between $T_{\max}$ and the temperatures measured with the other techniques; for lower *W* values, a gap appears and increases when *W* decreases. In the literature, it has been suggested that the melting temperature is given by the temperature $T_{\mathrm{end}}$ of the end of the exotherm, when the heat flow joins the baseline (Figure 4a) [17]. This definition is legitimate, since the liquidus is defined by the line above which the mixture is completely liquid, that is when the last solid fraction has melted. For instance, it is applied to map phase diagrams of metal alloys [14]. However, in the case of organogels, the heat flow broadens after the peak, especially for low concentration *W*. As a result, $T_{\mathrm{end}}$ values are well above the transition temperature values, as measured by other techniques (Figure 4b).

The tangent method is another classical method to measure the full solubilization temperature of organic compounds by DSC [21]. It consists of drawing the tangent at the inflection point and determining its intersection with the baseline (Figure 4a). This method is very precise for concentrated solution (a few tens of wt %) because the baseline is well defined. However, for lower concentrations, the baseline is not linear and noisy, which leads to large uncertainties. However, the inflection point can be located with accuracy and very reproducibly even for a heat flow with a low signal/noise ratio, as shown in Figure 4a. The temperature of full solubilization can be visualized by the integral of the heat flow. This integral varies linearly with the amount of gelator in the liquid phase (Equation (2)). At the plateau, the gelator is fully soluble and in the ascending branch increasing with *T*, the gelator is transforming from solid to liquid. The temperature of full solubilization can be taken as the intersection of the plateau and the tangent of the integral at its inflection. It is closer to the liquidus and to the $T_{\mathrm{GS}}$ measured by rheology than $T_{\max}$; it presents errors but less bias. The determination of the melting temperature by the integral is rather lengthy. However, as shown in Figure 4a,b, the values are close to the inflection points within 1 °C, and the inflection point is simpler to determine, either graphically or by software. Therefore, $T_{m}$ can be measured conveniently with a good precision by the inflection point temperature.

### 2.4. Calculation of the Soluble Fraction of Gelator from the Thermograms

**Figure 5:**
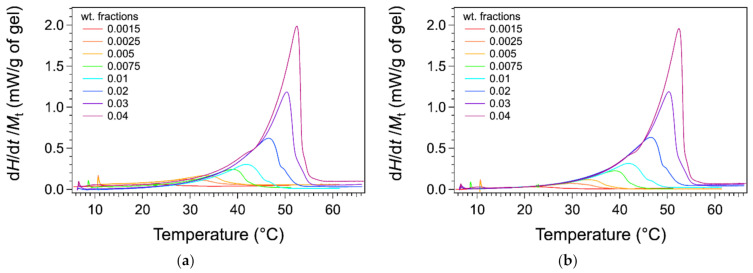
Thermograms normalized to the total mass of gel. (**a**) Raw data. (**b**) Thermograms corrected from the heat capacity differences of the cell as described in Materials and Methods. The ascending parts of the curves before transition temperatures $T_{m}$ are superimposed.

When the thermograms are normalized to the total mass of the gel, their ascending branches superimpose approximately (Figure 5a). In this section, we try to study whether this superimposition and the shape of the thermograms reflect the thermodynamics of the organogels. We have hypothesized that the heat flows are related only to the transformations of the gel at quasi-equilibrium. The baseline of the thermograms was corrected from the difference in capacity between cells, as described in Materials and Methods, which made the superimposition more accurate, as shown in Figure 5b.

After this correction of the baselines, the heat flows correspond to Equation (5).

(5) $$\frac{1}{M_{t}}\frac{dH}{dT}=\frac{L}{M}\frac{dW_{l}}{dT}+W_{l}\left(T\right)∆C_{p}$$

where $W_{l}$ is the weight fraction of solubilized gelator in the whole mixture, *M* is the molar weight of the gelator, *L* is the molar melting enthalpy, and $∆C_{p}$ is the difference between the heat capacity of the solid and liquid gelator. $W_{l}$ is close to the weight fraction $w_{l}$ of the gelator in the liquid (Equation (6)).

(6) $$w_{l}=\frac{W_{l}}{1-W+W_{l}}$$

When the gel is heated but not melted, the solubility $w_{l}\left(T\right)$ is equal the liquidus (Figure 3). Hence, when the heat flows are normalized to the total mass $M_{t}$, they depend only on intensive quantities. It explains why the curves superimpose before the melting of the gels. After the transition, $W_{l}$ = *W* and $dW_{l}/dT$ = 0, and the heat flows reach a plateau of different values.

In a first approximation, the capacity effects are neglected, and the heat flows are:

(7) $$\frac{dH}{dT}=\frac{LM_{t}}{M}\frac{dW_{l}}{dT}$$

The integration of the curves should yield the solubility and retrace the liquidus in the phase diagram. The weight fraction $W_{l}$ of the gelator in the mixture is expressed simply with $∆H_{1}^{T}$, the integral between a reference temperature $T_{1}$ and *T*, and ∆H is the maximal value of this integral (as defined in Section 2.3):

(8) $$W_{l}\left(T\right)=W-\frac{M}{LM_{t}}\left(∆H-∆H_{1}^{T}\right)$$

**Figure 6:**
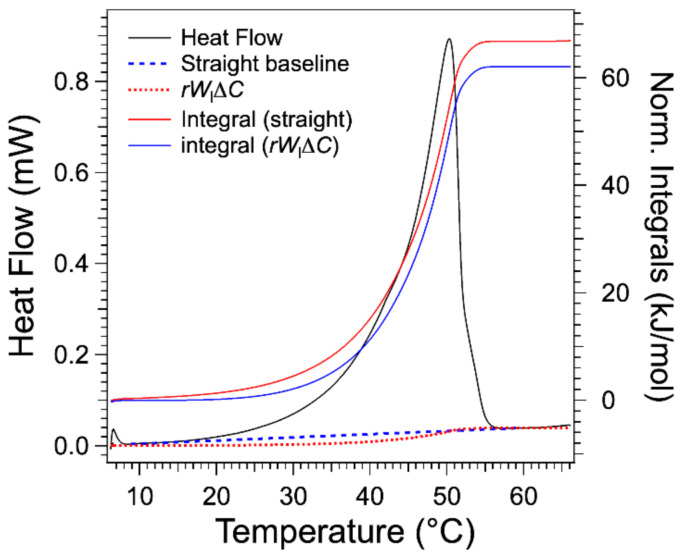
Thermogram of HSA/nitrobenzene (0.03 wt. fraction). Integration of the heat flow after subtraction of two different baselines. The straight baseline is the one subtracted from the commercial software. The other baseline is $rW_{l}∆C_{p}$ ($W_{l}$ calculated from Equation (8) and normalized to match the value of the plateau after the transition ≈$W∆C_{p}$ and multiplied by the heating rate *r* to convert the quantity to heat flow).

When the heat flow is integrated after the subtraction of a straight baseline joining the integration limits, the $w_{l}$ values yielded by Equations (4) and (6) superimpose with the liquidus only at high T and depart significantly from it at low T or concentrations. This error is due to the straight baseline that neglects the partial melting at low *T*, as explained in Section 2.2. In order to better estimate the baseline, $W_{l}\left(T\right)$ was multiplied by a factor to adjust the value of its plateau (*W*) to the baseline of the normalized heat flow after $T_{m}$. It amounts to multiplying by $∆C_{p}$, and we verified that the factor is close to the $∆C_{p}$ value calculated from the fit in material and method. Since Equation (5) deals with d*H*/d*T*,the conversion to the heat flow d*H*/d*t* is done by multiplying the resukt by another factor *r*, the heating rate. Figure 6 compares the term $rW_{l}∆C_{p}$ and the straight baseline for the gel at 3 wt %. It lies below the straight baseline joining both integral limits. Therefore, the integral after subtraction of this term yields a higher molar enthalpy of about 7% in the selected example, and this error increases when the concentration *W* decreases to reach more than 50% for the lowest concentrations. Indeed, the order of magnitude of the error is $w_{l,1}/W$. The integrals calculated after subtraction of the $rW_{l}∆C_{p}$ yield a value of molar melting enthalpy *L* of 67.1 ± 0.7 kJ/mol.

**Figure 7:**
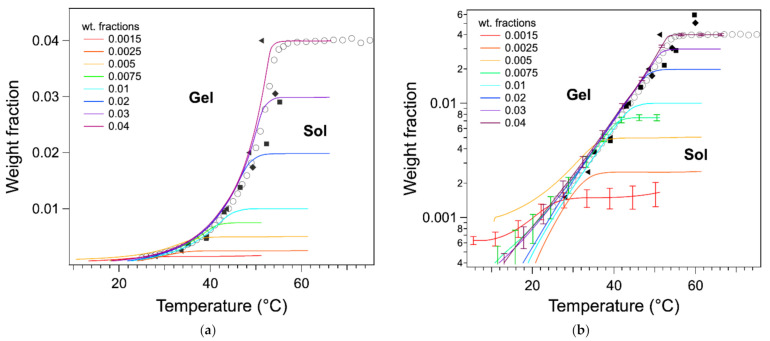
Derivation of the weight fractions $w_{l}\left(T\right)$ of HSA in the liquid phase from the integrals of the thermograms from Equations (5) and (6) and their superimposition with the phase diagram. (**a**) Linear representation; (**b**) logarithm representation. The different points are those of the phase diagram (Figure 4b, with switched axes) with the same legend. For clarity, errors bars have been represented only for three samples (0.0015, 0.0075, and 0.04) and every 400 points.

The ascending parts of the $w_{l}\left(T\right)$ curves superimpose well with the liquidus as measured by other techniques (Figure 7). For the sample of concentration *W* > 0.075, the curves superimpose from high to low temperatures within 2 °C in their upper parts. For the samples of concentration below 0.005, the curves superimpose only roughly to the phase diagram. Part of this error can be attributed to the low signal/noise ratio at lower *W* and the difficulty of calculating the baseline. The uncertainties of $w_{l}\left(T\right)$ were calculated by taking into account the noise, the errors on *W*, and the measured *L* values. For the curves corresponding to high concentrations, the uncertainties are below 10% for temperatures above 35 °C and increase when T increases. For lower concentrations, the uncertainties are greater than 10% even if it decreases with decreasing *T*. In addition to the errors due to the signal, and more fundamentally, at low temperature, the heat flows cannot be modeled only by thermodynamics at equilibrium, but the heat transfer and kinetics should be taken into account. However, this superimposition shows that the shape of the endotherms above ≈34 °C are directly related to the phase diagram. It also confirms that the determination of the melting temperature by the integrals is more accurate: the temperature of the maximum of the endotherm is too low because it corresponds to the inflection point of the integral, not to the start of the plateau.

### 2.5. Comparison of the Endotherms with the Exotherms

**Figure 8:**
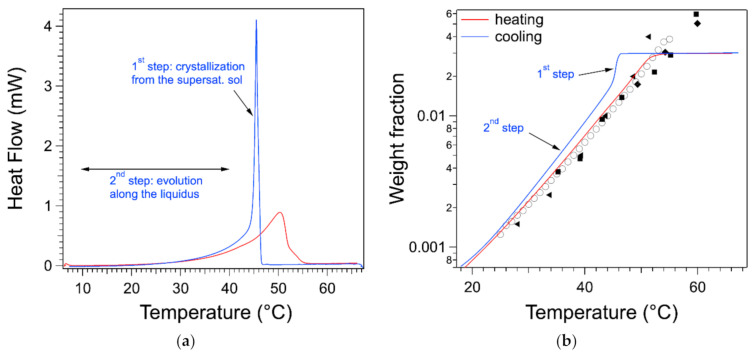
(**a**) Comparison of the heat flow during heating (red) and cooling (blue); *W* = 0.03. One of the thermograms is multiplied by −1 to make both flows positive and compare them. (**b**) Weight fractions of the gelator in the liquid phase derived from both heat flows with Equations (6) and (8). On cooling, the plateau crosses the liquidus, indicating a supersaturated solution. The drop in concentration indicates the solidification of the gelator.

As discussed in the introduction, the thermogram measured upon cooling exhibits a sharp peak. In Figure 8a, the endotherm and the exotherm of the gel at 0.03 are represented. The exotherm has been multiplied by −1 to compare directly both flows. On the cooling curve, after the heat flow reaches its peaks, when *T* decreases, the flow decreases first abruptly and then slowly. In the second step, at low temperatures, it superimposes with the heating curve. This can be explained by correlating the signal with the phase diagram.

The heat flow measured during cooling can be treated and integrated in the same way as the one during heating. It yields the variation of the weight fraction $w_{l}\left(T\right)$ during the cooling phase. $w_{l}\left(T\right)$ starts from the plateau where all of the gelator is soluble. When *T* decreases, $w_{l}\left(T\right)$ remains on the plateau, which shows that all of the gelator is in the liquid phase and passes the liquidus: the gelator remains dissolved but in a supersaturated state. At the transition temperature (almost equal to the peak temperature), the concentration drops suddenly. In a second stage, it decreases slowly with *T*, and it is close to the liquidus. The drop in $w_{l}\left(T\right)$ corresponds to the solidification of part of the gelator. This stops when the solution returns to its saturation state, i.e., on the liquidus. This return is slow and incomplete. It can be due to the kinetics of formation of the aggregates. Nevertheless, the heat flow shows both steps, as indicated on Figure 8a. The whole signal includes both stages, and the integral of the peak itself is only half of the whole integral. Therefore, integration limits close to the edges of the peak will yield erroneously a lower enthalpy than for the endotherm.

## 3. Conclusions

We have studied by microDSC the transitions of an organogel with a well-established phase diagram. The heat flow is proportional to the variation of the fraction of solubilized gelator with temperature. This quantity is linked to the shape of the liquidus (it is the inverse of the liquidus slope). The integrals of the heat flows, after suitable correction and normalization, follow the liquidus below the transition and reach a plateau after the transition. This relation is valid for temperatures above a threshold of 34 °C. In this regime, the thermograms can be predicted by the phase diagram only. Below the limit and especially for the samples at low concentrations, the integrals follow only approximately the phase diagrams. The heat transfer and kinetics are no longer negligible and probably broaden the endotherms after the transition. In order to quantify more precisely the shape of the endotherms, it will be necessary to model these non-equilibrium phenomena.

The relationship between heat flows and phase diagrams explains why the endotherms can be so stretched below the transition. It also provides hints to measure more precisely the melting temperature of a gel. This temperature can be measured precisely by the integrals and more readily with a good approximation at the inflection point following the peak.

We also find that classical integration of the heat flow, with a straight baseline, underestimates the value of the molar melting enthalpy *L*, because it neglects the dissolution at lower temperature.

The exotherms measured during cooling phases have usually sharper peaks, which corresponds to the sudden formation of the solid phase of the gel from the supersaturated sol. After the peak, when the temperature decreases further, the heat flows is governed by the liquidus, as in the heating phase. The integration must take into account this second step.

## 4. Materials and Methods

### 4.1. Materials

12-hydroxystearic acid (racemic) was purchased from Aldrich and used as is. Trans-Decalin (TCI) was purified prior to use by filtration on silica.

### 4.2. Micro-Differential Scanning Calorimetry

The thermograms were recorded with a SETARAM III microcalorimeter. The measuring cell was filled with a gelator/solvent mixture (between 100 and 200 mg). The reference cell was filled with a mass of pure solvent equal to that of the first cell within 0.1 mg. The gel was formed during a first cycle of heating at 1 °C/min and cooling at 0.3 °C/min. The gels were allowed to rest at 10 °C for 1 h, and the thermograms were measured upon heating at 0.1 °C/min. Another thermogram was measured when the temperature was cooled back at a set rate r = −0.10 °C. We checked that the effective rate *r* was constant throughout the measurement and equal to the set rate within 2%. The areas of the signals and the different temperatures as described in Figure 4a were calculated with homemade macros in Wavemetric Igor. The noise of the thermograms had a RMS of 0.7 µW and the peak-to-peak amplitudes comprised between 1 and 2 µW. The signal-to-noise ratios are comprised between 35 (0.0025) and 890 (0.03). RMS values of the noise and the errors on the weighed masses of the gelator and solvent were taken into account to estimate the errors of the melting enthalpies and calculated solubilities from the thermograms.

### 4.3. Correction of the Heat Flows

The heat flows were corrected from the cell capacities with the following procedure: The heat flows were first normalized to the total mass of gels $M_{t}$ (Figure 5a).

The normalized heat flow is given by Equation (9):

(9) $$\frac{1}{M_{t}}\frac{1}{r}{\left(\frac{dH}{dt}\right)}_{T}=\frac{L}{M}{\left(\frac{dW_{l}}{dT}\right)}_{T}+\frac{∆C_{\mathrm{cell}}}{M_{t}}+WC_{p,s}+W_{l}∆C_{p}$$

where *r* is the heating rate, $∆C_{\mathrm{cell}}$ is the difference of capacity between the reference and sample cell, $C_{p,s}$ is the massic heat capacity of the solid gelator, and $∆C_{p}$ is the difference of the heat capacities of the solid and liquid gelator.

$∆C_{\mathrm{cell}}$ and $WC_{p,s}$ are constants with *T* but vary from one sample to the other. The first two terms vary with *T*, but for a common temperature $T_{1}$, below the transitions, they have the same values. When *T* is above the melting temperature $T_{m}$, all the gelator is melted and the first enthalpic term vanishes, since $dW_{l}/dT$ is zero and $W_{l}=W$. We chose $T_{1}$ = 18 °C as a reference temperature below all the observed melting temperatures. The difference between the normalized heat flow at $T_{1}$ and the same flow above the melting temperature is:

(10) $$\begin{array}{cc} \delta \left(T_{1}\right)= & \frac{1}{M_{t}}{\left(\frac{dH}{dt}\right)}_{Hi}-\frac{1}{M_{t}}{\left(\frac{dH}{dt}\right)}_{T_{1}}=-K\left(T_{1}\right)+W∆C_{p}r\\ & \mathrm{with}\;\;K\left(T_{1}\right)=\frac{Lr}{M}{\left(\frac{dW_{l}}{dT}\right)}_{T_{1}}+W_{l,1}∆C_{p}r \end{array}$$

**Figure 9:**
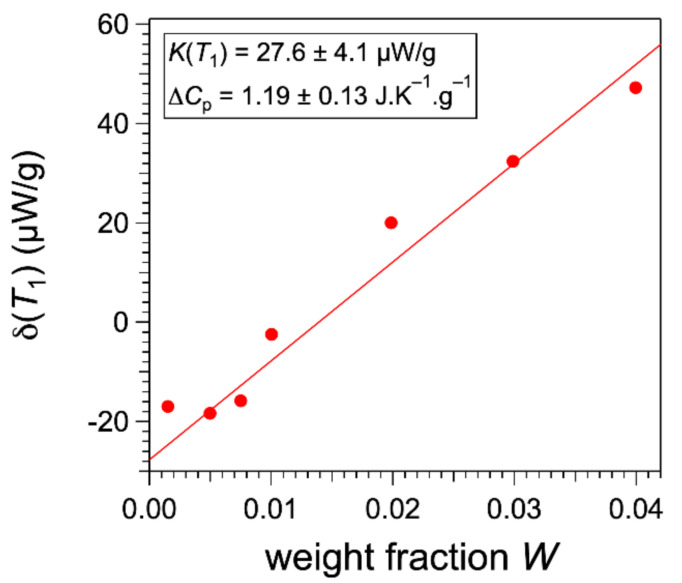
Variation of the gap $\delta \left(T_{1}\right)$ with *W* and measurement of constant $K\left(T_{1}\right)$.

In this equation, the first term −$K\left(T_{1}\right)$ is constant for all the curves, while the second one varies linearly with W. $\delta \left(T_{1}\right)$ is calculated for all curves plotted as a function of *W*, and the constant $K\left(T_{1}\right)$ is determined from the intercept with the *y*-axis (Figure 9).

Once the constant is determined, for each curve, the following corrective $\delta_{corr}$ term can be calculated and subtracted.

(11) $$\delta_{corr}=\frac{1}{M_{t}}{\left(\frac{dH}{dt}\right)}_{T_{1}}-K\left(T_{1}\right)=r\frac{∆C_{\mathrm{cell}}}{M_{t}}+rWC_{p,s}$$

## Data Availability

The data presented in this study are available on request from the corresponding author. The data are not publicly available due to lack of dedicated server.

## References

[1] Terech P., Weiss R.G. (1997). Low Molecular Mass Gelators of Organic Liquids and the Properties of Their Gels. Chem. Rev..

[2] Weiss R.G., Terech P. (2006). Molecular Gels. Materials with Self-Assembled Fibrillar Network.

[3] Hirst A.R., Escuder B., Miravet J.F., Smith D.K. (2008). High-Tech Applications of Self-Assembling Supramolecular Nanostructured Gel-Phase Materials: From Regenerative Medicine to Electronic Devices. Angew. Chem. Int. Ed..

[4] Babu S.S., Praveen V.K., Ajayaghosh A. (2014). Functional π-Gelators and Their Applications. Chem. Rev..

[5] Du X., Zhou J., Shi J., Xu B. (2015). Supramolecular Hydrogelators and Hydrogels: From Soft Matter to Molecular Biomaterials. Chem. Rev..

[6] Weiss R.G. (2018). Molecular Gels: Structure and Dynamics.

[7] Feng L., Cavicchi K.A. (2012). Investigation of the relationships between the thermodynamic phase behavior and gelation behavior of a series of tripodal trisamide compounds. Soft Matter.

[8] Kristiansen M., Werner M., Tervoort T., Smith P., Blomenhofer M., Schmidt H.-W. (2003). The Binary System Isotactic Polypropylene/Bis(3,4-dimethylbenzylidene)sorbitol:  Phase Behavior, Nucleation, and Optical Properties. Macromolecules.

[9] Kristiansen P.M., Gress A., Smith P., Hanft D., Schmidt H.-W. (2006). Phase behavior, nucleation and optical properties of the binary system isotactic polypropylene/N,N′,N″-tris-isopentyl-1,3,5-benzene-tricarboxamide. Polymer.

[10] Cavicchi K.A., Pantoja M., Lai T.-Y. (2018). The Importance of Phase Behavior in Understanding Structure-Property Relationships in Crystalline Small Molecule/Polymer Gels. Gels and Other Soft Amorphous Solids.

[11] Raghavan S.R., Cipriano B.H., Weiss R.G., Terech P. (2006). Gel Formation: Phase Diagrams Using Tabletop Rheology and Calorimetry. Molecular Gels: Materials with Self-Assembled Fibrillar Networks.

[12] Terech P., Rossat C., Volino F. (2000). On the Measurement of Phase Transition Temperatures in Physical Molecular Organogels. J. Coll. Interf. Sci..

[13] Toro-Vazquez J.F., Pérez-Martínez J.D. (2018). Chapter 3: Thermodynamic Aspects of Molecular Gels. Molecular Gels: Structure and Dynamics.

[14] Zhao J.-C. (2011). Methods for Phase Diagram Determination.

[15] Menger F.M., Caran K.L. (2000). Anatomy of a Gel. Amino Acid Derivatives That Rigidify Water at Submillimolar Concentrations. J. Am. Chem. Soc..

[16] Desvergne J.-P., Brotin T., Meerschaut D., Clavier G., Placin F., Pozzo J.-L., Bouas-Laurent H. (2004). Spectroscopic properties and gelling ability of a set of rod-like 2,3-disubstituted anthracenes. New J. Chem..

[17] Guenet J.-M. (2016). Organogels: Thermodynamics, Structure, Solvent Role, and Properties.

[18] Kousksou T., Jamil A., Zeraouli Y., Dumas J.-P. (2007). Equilibrium liquidus temperatures of binary mixtures from differential scanning calorimetry. Chem. Eng. Sci..

[19] Christ E., Collin D., Lamps J.-P., Mésini P.J. (2018). Variable temperature NMR of organogelators: The intensities of a single sample describe the full phase diagram. Phys. Chem. Chem. Phys..

[20] Christ E., Blanc C., Al Ouahabi A., Maurin D., Le Parc R., Bantignies J.-L., Guenet J.-M., Collin D., Mésini P.J. (2016). Origin of Invariant Gel Melting Temperatures in the c–T Phase Diagram of an Organogel. Langmuir.

[21] Mohan R., Lorenz H., Myerson A.S. (2002). Solubility Measurement Using Differential Scanning Calorimetry. Ind. Eng. Chem. Res..

